# SARS-CoV-2 vaccines: What we know, what we can do to improve them and what we could learn from other well-known viruses

**DOI:** 10.3934/microbiol.2022029

**Published:** 2022-11-16

**Authors:** Sirio Fiorino, Andrea Carusi, Wandong Hong, Paolo Cernuschi, Claudio Giuseppe Gallo, Emanuele Ferrara, Thais Maloberti, Michela Visani, Federico Lari, Dario de Biase, Maddalena Zippi

**Affiliations:** 1 Internal Medicine Unit, Budrio Hospital, Budrio (Bologna), Azienda USL, Bologna, Italy; 2 Department of Gastroenterology and Hepatology, First Affiliated Hospital of Wenzhou Medical University, Wenzhou City, Zhejiang, The People's Republic of China; 3 Internal Medicine Unit, Quisana Private Hospital, Ferrara, Italy; 4 Emilian Physiolaser Therapy Center, Castel S. Pietro Terme, Bologna, Italy; 5 Internal Medicin Unit, IRCCS S.Orsola Hospital, Bologna, Italy; 6 Department of Experimental, Diagnostic and Specialty Medicine, University of Bologna-Molecular Diagnostic Unit, Azienda USL di Bologna, Bologna, Italy; 7 Department of Pharmacy and Biotechnology, University of Bologna, Bologna, Italy; 8 Solid Tumor Molecular Pathology Laboratory, IRCCS Azienda Ospedaliero-Universitaria di Bologna, Bologna, Italy; 9 Unit of Gastroenterology and Digestive Endoscopy, Sandro Pertini Hospital, Rome, Italy

**Keywords:** SARS-CoV-2, vaccine, virus, review

## Abstract

In recent weeks, the rate of SARS-CoV-2 infections has been progressively increasing all over the globe, even in countries where vaccination programs have been strongly implemented. In these regions in 2021, a reduction in the number of hospitalizations and deaths compared to 2020 was observed. This decrease is certainly associated with the introduction of vaccination measures. The process of the development of effective vaccines represents an important challenge. Overall, the breakthrough infections occurring in vaccinated subjects are in most cases less severe than those observed in unvaccinated individuals. This review examines the factors affecting the immunogenicity of vaccines against SARS-CoV-2 and the possible role of nutrients in modulating the response of distinct immune cells to the vaccination.

## 1. Introduction

In recent weeks, the rate of severe acute respiratory syndrome coronavirus 2 (SARS-CoV-2) infections has been progressively increasing all over the globe, even in countries where vaccination programs have been strongly implemented. These regions, in 2021, observed a reduction in the number of hospitalizations and deaths compared to 2020. This decrease is certainly associated with the introduction of vaccination measures since the beginning of 2021. However, the start of the autumn season, as was foreseeable [1], has been characterized by a new rise in SARS-CoV-2 infection rates in the global population. As it has been known since the Hippocratic era, the incidence of respiratory infectious diseases is influenced by weather conditions [2] and follows seasonal patterns with high incidence during winter in temperate regions and during the rainy season in tropical regions [3]. The decrease in solar radiation during the autumn and winter seasons, as well as the reduction in temperature, variations in relative humidity of the air and the presence of atmospheric pollutants, are associated with the strong resumption of infections from SARS-CoV-2 in individuals living in Western European nations [1]. The hospitalizations and deaths were observed mainly in unvaccinated subjects [4]–[6].

### 1.1. Considerations on the feasibility of developing effective vaccines against SARS-CoV-2 in a short time

The availability of vaccines effective in counteracting the microorganisms invading humans represents the most useful strategy for the prevention of infectious diseases. In particular, this approach mainly relies on the induction of an adaptive immune response against pathogens, and it has contributed to limiting the spread of some life-threatening viruses in humans, resulting in either their eradication (smallpox virus) or the control of their diffusion worldwide (poliomyelitis and hepatitis B virus (HBV)) [7]. However, the process for the development of effective vaccines represents an important challenge, as these preparations have to exhibit adequate strength, quality and safety to the generation of efficient and protective immunity. Considerations when developing an effective and safe vaccine may be summarized as follows: i) the potential poor immunogenicity or instability of the proteins incorporated into the vaccines, ii) in case of vaccines with live attenuated microorganisms, the risk of conversion into more virulent forms, iii) the need for binding adjuvant substances to inactivated infectious agents to stimulate the immune response and iv) the development of possible mutations in the genome of pathogens, i.e., mainly viruses, during their diffusion in distinct geographical areas and different populations [8]. In particular, the latter point represents a critical topic. The development of vaccines with the capability of counteracting viruses has to take into account the characteristics of these pathogens, such as their structure, their genome organization and the mechanisms controlling their replication. In particular, all of these factors have made difficult the preparation of vaccines against RNA viruses. Despite numerous efforts, to date, this preventive strategy is not available in clinical practice for at least two RNA viruses, causing a heavy disease burden in mankind worldwide: human immunodeficiency virus (HIV) and hepatitis C virus (HCV). Several reasons contribute to explaining the difficulty in creating effective vaccines against RNA viruses. In particular, a large series of structural and non-structural components of these pathogens, as well as some pathways and mechanisms regulating their cycle life, may have an important impact on the preparation of these products. Replication is a crucial process in RNA virus biological behavior, and the RNA-dependent RNA polymerase enzyme exerts a key role in this event. The activity of this protein is associated with two key challenges in developing a vaccine: a) an elevated viral replication rate; b) a high error rate during viral transcription, as this enzyme lacks proofreading activity capability and post-replicative RNA repair mechanisms. Therefore, millions of copies of the viral genome are generated in each infected host every day, and this event is associated with the emergence of a very large spectrum of variants. Although most of these display a decreased fitness that absolutely prevents replication or leads to their extinction, a small fraction of them may escape from the pressure due to the immune system, specific pharmacological treatments and vaccines [9]. Overall, the following characteristics of RNA-virus subfamilies have contributed to limiting the development of vaccines against these pathogens: 1) different, but closely related, variants known as “quasispecies”; 2) different genotypes and subtypes, identified according to genotyping analysis; 3) current unavailability of immunological tests to accurately predict protection and test the efficacy of new vaccines against emerging viruses in humans [10]. At the end of 2019, a new coronavirus defined as SARS-CoV-2 emerged in Wuhan, China, and it has quickly spread worldwide. The pathological condition induced by this pathogen is known as COVID-19, and the pandemic associated with this virus has been defined as a “public health emergency of international concern” by the International Health Regulation Emergency Committee of the World Health Organization (WHO) [1]. Since then, important efforts have been performed to make available valid strategies to counteract SARS-CoV-2 related infection. Like all other RNA viruses, SARS-CoV-2 also presents a high rate of genome mutations during each cycle of replication. The generation of these viral strains may confer a survival advantage to this pathogen, potentially increasing its capability of spreading and exerting its pathogenetic effects.

Among the possible preventive interventions, vaccination is also considered as an effective routine measure for the control of this pathogen, and this approach has received a significant boost in the past year of the pandemic. Several novel vaccines against SARS-CoV-2 have been approved for clinical use (Table 1), whereas other preparations are currently being tested as pre-clinical or clinical candidates.

To date, the spike (S) protein represents the primary target for the majority of these products. These preparations stimulate the generation of protective antibodies with neutralizing activity against this pathogen in the host. The currently available platforms of anti-SARS-CoV-2 vaccines include a) mRNA-based ones, such as mRNA BNT162b2 (Pfizer-BioNTech/Comirnaty, available in the USA, Europe and the United Kingdom) and mRNA-1273 (Moderna, available in the USA, Europe and the United Kingdom), b) adenoviral vector-based ones, such as Ad26.COV2.S (Johnson & Johnson/Janssen, available in the USA and Europe), AZD1222 (Oxford-AstraZeneca/Vaxzevria, available in Europe and the United Kingdom) and Gam-COVID-Vac (Sputnik V, Russian Ministry of Health), and c) inactivated ones, such as CoronaVac (Sinovac Biotech) and WIBP / BBIBP-CorV COVID-19 (Sinopharm), National Medical Products Administration, China.

**Table 1.** Currently available platforms of anti-SARS-CoV-2 vaccines.

Vaccine	Technology	Manufacturer	Availability
mRNA BNT162b2	mRNA based	Pfizer-BioNTech/Comirnaty	USA, Europe, and U.K.
mRNA-1273	mRNA based	Moderna	USA, Europe, and U.K.
Ad26.COV2.S	adenoviral vector-based	Johnson & Johnson/Janssen	USA and Europe
AZD1222	adenoviral vector-based	Oxford-AstraZeneca/Vaxzevria	Europe and UK
Gam-COVID-Vac	adenoviral vector-based	Sputnik V	Russia
CoronaVac	inactivated	Sinovac Biotech	Unknown
WIBP/BBIBP-CorV COVID-19	inactivated	Sinopharm	Unknown

### 1.2. Current knowledge of available anti-SARS-CoV-2 vaccines and potential concerns about the efficacy against this pathogen

Like other viruses, and since the beginning of the current pandemic, several genetic lineages harboring a large series of mutations have progressively emerged in the genome of the original causative agent of COVID-19 and have been detected and described worldwide [11]. The accumulation of changes in nucleotide sequences of SARS-CoV2 RNA is associated with amino acid substitutions in viral proteins and the generation of variants, differing from the “wild-type” virus [12]. This process is defined as “antigenic drift”, and the emergence of these different viral lineages may have an important impact on human health, leading, for example, to i) increased transmissibility, morbidity and mortality; ii) decreased capability to detect them by available diagnostic tests, with a potential delay in the diagnosis and treatment; iii) reduced susceptibility to antiviral therapies, such as antiviral drugs, monoclonal antibodies and convalescent plasma; iv) higher risk of reinfection in previously infected and recovered subjects; and v) development of vaccine breakthrough cases [13]. To date, beginning after their identification, some of these different variants are routinely monitored by the USA's SARS-CoV-2 Interagency Group due to their potential elevated risk to public health. According to the Pango classification, these variants include B.1.1.7, B.1.351, P.1, B.1.617.2 and B.1.1.529 B. Recently, the WHO has established a simple way to identify the key lineages of SARS-CoV-2 by using letters of the Greek alphabet. Therefore, the currently described different variants are defined as Alpha (B.1.1.7), Beta (B.1.351), Gamma (P.1), Delta (B.1.617.2), Epsilon (B.1.427 and B.1.429), Zeta (P.2), Eta (B.1.525), Theta (P.3), Iota (B.1.526), Kappa (B.1.617.1), Lambda (C 37), Mu (B.1.621, B.1.621.1) and Omicron (B.1.1.529) ones (https://www.cdc.gov/coronavirus/2019-ncov/variants/variant-info.html). Furthermore, the WHO has indicated the different SARS-CoV-2 variants of interest (VOIs), variants of concern (VOCs) and variants of high consequences (VOHC) on the basis of their features and attributes that potentially increase the risk to global human health and require public health intervention. Therefore, on the basis of this definition, Lambda and Mu lineages have been included in the first group (VOI), whereas Alpha (B.1.1.7), Beta (B.1.351), Gamma (P.1), Delta (B.1.617.2) and Omicron (B.1.1.529) ones have been classified as part of the second group (VOCs) (Table 2).

**Table 2.** SARS-CoV-2 variants.

Lineage	Type of Variant
Lambda	VOI
Mu	VOI
Alpha (B.1.1.7)	VOCs
Beta (B.1.351)	VOCs
Gamma (P.1)	VOCs
Delta (B.1.617.2)	VOCs
Omicron (B.1.1.529)	VOCs

VOI: variant of interest; VOCs: variants of concern

On the other hand, to date, no variants of high consequence have been identified. The earliest reports have stated that the majority of available vaccines (mRNA-BNT162b2 and mRNA-1273, Ad26.COV2.S and AZD1222) are generally well tolerated and capable of preventing the development of symptomatic cases, the need for intensive care unit (ICU) admission and the risk of death in most of the vaccinated individuals. The criteria for defining effective vaccines against SARS-CoV-2 have been summarized in a recent review [14]. The available trials have shown that the efficacy of this preventive measure is variable and that it ranges from 70.4% (AZD1222) to 90–95% (mRNA BNT162b2 and mRNA-1273). In particular, according to current evidence, mRNA vaccines may induce a robust production of IgG class antibodies with neutralizing activity toward SARS-CoV-2, eliciting robust antiviral responses from 7 to 14 days after the administration of the second dose. Some concerns have emerged in recent months, as a breakthrough infection has also been observed in vaccinated individuals. According to the current evidence, the protection against the risk of hospital admission and death generally remains elevated in persons who have completed the cycle of vaccine administration [15]. Overall, the breakthrough infections occurring in these subjects are, in most cases, less severe than those observed in unvaccinated individuals [16]. Nevertheless, hospitalizations, symptomatic diseases and deaths are slowly, but progressively, increasing worldwide even among vaccinated people [17],[18]. RNA-based vaccines are effective in individuals with a high risk of SARS-CoV-2 infection in the real life; however, the protection rates observed in these subjects are low as compared to ones previously reported. Cohabitants of individuals suffering from COVID-19 and subjects with significant exposure to SARS-CoV-2 still display a significant risk of infection even if they have been fully vaccinated [19]. The genesis of the reduction of vaccination effectiveness is still a matter of debate and may be due to different reasons. However, the development of SARS-CoV-2 variants (particularly the Delta variant) and/or the progressive decrease in neutralizing serum antibody titers represent probable causes [20]. It is likely that, in addition to the humoral arm of the immune response, the T cell compartment, with the development of memory T cell clones, exerts a hallmark role in the achievement of the protective immune response after acute infection, as well as after an effective vaccination.

A recent trial has shown that the humoral response substantially begins to drop after approximately 5–6 months after the completion of the BNT162b2 vaccination cycle, and this decline mainly occurs in men, people older than 65 years and individuals with immunosuppression [21].

Aged, immunosuppressed and poly-pathological patients are at the greatest risk of severe forms of illness associated with SARS-CoV-2 infection, with a high probability of required hospitalization and intensive care support, as well as death, in comparison with younger and healthier individuals [22]. Therefore, based on this observation, since October 2021, the WHO and the Center for Disease Control and Prevention (USA) have suggested that effective and adequate anti-SARS-CoV-2 protection may be retrieved via the administration of a booster dose of the vaccine [23],[24]. According to this assumption, some nations, including Israel and several European countries, have started additional vaccination programs for their inhabitants [25]. On the basis of the results available in scientific literature, this strategy seems to restore valid antiviral protection. Some problems persist, as the duration of this novel defensive measure and its effectiveness against the different SARS-CoV-2 variants are unclear. Furthermore, a threshold titer of antibodies against the spike protein (SP) useful to predict the recurrence of the infection has not yet been defined [26].

## 2. Immune system activity against SARS-CoV-2 in infected and vaccinated individuals

The most recent pieces of evidence suggest that the protection against SARS-CoV-2 also depends on the well-coordinated and properly regulated activity between innate and adaptive arms of the immune system, as it has already been demonstrated for other known human viral infectious diseases [27]. Macrophages and dendritic cells play a crucial role in orchestrating the early phase of defensive response against viruses. These antigen-presenting cells (APCs), as part of the innate arm of the immune system, capture the viral capsids, process their constitutive proteins and expose their fragments (defined as epitopes) on their cell membrane [28]. This event is associated with the stimulation of the adaptive component of the immune response via the involvement of additional cell subsets. T helper cells (CD4+) recognize specific viral epitopes and release interleukins, cytokines and other mediators [29]. Overall, the coordinated activation of this process boosts both humoral and cellular adaptive specific antiviral responses and may lead to the efficient control of these pathogens [30]. Since the beginning of the pandemic, research has been focused on investigating the immune processes involved in the host's defense against SARS-CoV-2 and its VOCs [31]. A detailed description of the host's immune mechanisms and functions that take part in the control of this pathogen is beyond the purpose of this paper. The early studies on the defensive responses detectable in individuals with COVID-19 have shown that, as in other viral-related diseases, the immune system may effectively counteract SARS-CoV-2 infection via the tightly regulated activation of the host's innate and adaptive arms [32]. In particular, the cooperation among the components of innate immunity, such as the molecules of the interferon (IFN) system, the neutrophils/macrophages/natural killer/APCs and the elements of the adaptive one, including the different subsets of T and B cells, represents a pivotal event in the defense against SARS-CoV-2 [33],[34]. Current evidence suggests that IFN type I exerts an effective anti-SARS-CoV-2 role and contributes to the control of its replication and ability to induce disease in infected people [35]. Furthermore, asymptomatic SARS-CoV-2-positive individuals or subjects with mild forms of COVID-19 are able to mount effective and rapid virus-specific humoral and cellular immune responses [36],[37]. On the other hand, the dysfunctional interplay among IFN system molecules/neutrophils/APCs/macrophages, as well as lymphocytes B and T, have been associated with the development of severe COVID-19 forms [38],[39]. So far, most studies concerning the adaptive arm of the immune response against this pathogen and its variants have mainly focused on examining its humoral component and the analysis of antibodies produced either during SARS-CoV-2 infection or those induced by vaccination. Only a limited number of trials have investigated in depth the dynamic characteristics and functions of the T lymphocytes involved in the immune response toward this virus [40]–[45].

Recently, researchers have been directing their efforts to the achievement of more accurate knowledge about the anti-SARS-CoV-2 role of different subtypes of T lymphocytes. The current experimental and clinical studies confirm that the T cell compartment of the immune system, in cooperation with innate and humoral components of the immune response, represents a cornerstone in the host's defense against this pathogen [46]. This assumption was derived from some interesting evidence: i) T cells are able to control SARS-CoV-2 infection even if B cell responses are insufficient, as has been reported in two X-linked agammaglobulinemia patients suffering from pneumonia caused by this pathogen [47]. ii) Marked T lymphopenia is detectable in individuals with severe forms of COVID-19 [48]. The presence of high frequencies of some T lymphocyte subclasses, such as Th17 or T regulatory cells, has been correlated with a higher risk of disease severity and a poor outcome [49],[50]. Only recently, the investigations have been suggesting that the achievement of a longitudinal protective immunity in COVID-19 convalescents and vaccinated subjects, as well as the development of long-term protection upon re-exposure to this pathogen both in the single individual and in the general population, depends on a coordinated, specific and polyclonal immune response, mediated by CD4/CD8 T- and B-lymphocytes, toward a wide spectrum of different epitopes belonging to distinct SARS-CoV-2 proteins [51]. This scenario had already been observed in the past when the progressive improvement in technology had led to the introduction of cutting-edge investigation methods such as HLA-peptide tetrameric complex analysis. These innovative strategies have supported the ex vivo study of T lymphocyte kinetics and functionality in patients suffering from several types of virus-related infections, including HIV, HCV and HBV [52]–[56].

## 3. Possible similarities in the phenotypic characteristics and dynamic activity of CD4+ and CD8+ T cells in patients with SARS-CoV-2 acute disease and other viral infections

The study of the role of CD4+ and CD8+ T lymphocyte clones in patients with acute or persistent HBV infection may provide useful lessons and insight to increase knowledge on immunological events occurring in SARS-CoV-2-positive subjects. Moreover, this evidence can help to characterize which responses of the immune system are either protective or harmful in COVID-19 patients, with the intention of improving the search for valid drugs against this virus and the design of more effective vaccines for it [1]. The results of this comparison may summarize and recapitulate the types of anti-SARS-CoV-2-specific T cell clones, as well as the spectrum of innate and adaptive responses detectable in patients with this infection [57].

The so-called “heterologous immune response” [53],[57]–[60] identifies two distinct types of T cell-mediated responses, including both antigen-dependent and antigen-independent T cell stimulation. These events are mediated either by cross-reactive memory T cells, responding to antigens that differ from the ones initially presented, or by T cells specific to antigens of infectious agents that have previously invaded the host, which are reactivated in the presence of unrelated pathogens [61]. This latter phenomenon is known as bystander activation, and it is triggered in a TCR-independent and cytokine-dependent way in the presence of Type I IFNs, interleukin (IL)-18 and IL-15 [62]. Recent studies have shown that this event may not only affect CD8+ T cells, but also CD4+ T lymphocytes [63]. However, bystander activation of CD8+ T lymphocytes is better studied, and it may display a dual role, exerting either an overall protective function in the early stages of infections, or a harmful effect, and it may cause collateral damage to the host. It has been suggested that this phenomenon is a primary line of the host's defense, as it develops quickly after innate cytokine release (e.g., Type I IFNs, IL-18 and IL-15), which is before the induction of an adaptive response specific to the invading microorganisms. Therefore, this event promotes the generation of an inflammatory process with the recruitment of immune cells to the site of infection and it contributes to the control of the pathogens [64]–[66].

Peripheral blood mononuclear cells obtained from patients with other viral infections, such as HBV, and depleted of CD4+ CD25 + reg T lymphocytes release growing levels of IFN-γ after stimulation with viral antigens [67]. Individuals with acute or severe persistent HBV present an increase in circulating Th17 cells relative to healthy controls and patients with mild chronic hepatitis [68]. Therefore, it may be useful to carefully examine the data concerning the available investigations into the activity of the immune system in patients with other viral infections, such as acute or persistent HBV infection, and to compare them with the results of the early studies on innate and adaptive immune responses from individuals suffering from COVID-19. This strategy may provide proper insight and lessons, leading to the identification of possible common immune responses and mechanisms involved in the pathogenesis of both infections and contributing to the development of new drugs and vaccines against SARS-CoV-2.

Despite very recent efforts, our knowledge in this field of research is still limited, and a better understanding of the spectrum, structure and generation pathway of SARS-CoV-2 epitopes, as well as of the capability of this pathogen to stimulate T and B lymphocytes responses in the different phases of infection, is required. The lack of definitive conclusions may derive from the still limited number of studies that globally evaluate the response of the immune system to all proteins comprising SARS-CoV-2, and from the small size of the analyzed case series.

Some investigations have been performed or are in progress to assess not only the phenotypic characteristics, but also the dynamic changes in the activity of the T and B cells specific to patients suffering from COVID-19 [37]. A significant T-cell lymphopenia represents a key feature in patients with severe COVID-19 as compared to mild or moderate forms of SARS-CoV-2 disease [48],[69]. A similar finding has been observed in patients suffering from acute viral hepatitis B since the 1980s. In particular, the peripheral T lymphocyte count was significantly decreased in individuals who died from the disease as compared to subjects who recovered [70]. Furthermore, current studies are also examining SARS-CoV-2 and its VOCs, which have distinct viral epitopes belonging to different proteins of this pathogen that may be recognized by specific T lymphocytes, as well as how they may elicit the host's immune response both in infected and vaccinated individuals [39],[71]. In the 1990s, similar procedures and methodologies made it possible to detect immunodominant epitopes on different HBV proteins that are capable of eliciting specific CD4/CD8 T cell responses against this pathogen [72]–[74]. To date, SARS-CoV-2-specific T cell peptides containing epitopes of this pathogen have been identified in several studies in convalescent subjects who had COVID-19, including fragments from the spike, from the M, from the NP and the ORF proteins [34],[75]. In particular, through the use of HLA class I and II predicted peptides, “mega pools” circulating SARS-CoV-2-specific CD8+ and CD4+ T lymphocytes have respectively been found in 70% and 100% of COVID-19 convalescent individuals. In this study, CD4+ T cells recognized a broad spectrum of viral proteins, including M, spike and N proteins, each representing about 11–27% of the total CD4+ responses, as well as nsp3, nsp4, ORF3a and ORF8. On the other hand, CD8+ T cells targeted spike, M and at least eight SARS-CoV-2 ORFs. Furthermore, SARS-CoV-2-reactive CD4+ T cells were identified in about 40–60% of subjects unexposed to the virus [76]. Overall, all of these data suggest that, as observed in individuals with acute limited HBV infection, patients who recover from COVID-19 or who undergo immunization after vaccination are able to mount a strong polyclonal and multispecific T cell immune response against a large series of SARS-CoV-2 antigens. To date, a large amount of evidence has demonstrated that epitopes recognized by CD8 T cells from COVID-19 patients are largely localized outside of the SP [77]. However, several studies have been focused on the immune response elicited by the viral SP and on the distinctive features and functions of CD4/CD8 T lymphocytes subsets, which target specific epitopes of this protein and which have been obtained from longitudinal specimens of patients suffering from COVID-19 with different levels of severity, ranging from mild illness to severe, even including death [78]. In a study trial, Neidleman and colleagues performed a phenotypical analysis of SARS-CoV-2-specific T cells, i.e., both CD4+ and CD8+ ones, from a small number of patients who have recovered from mild COVID-19. CD4+ T clones specific to this pathogen, which have been isolated from these subjects, possessed several peculiar characteristics. In particular, these lymphocytes presented the features of central memory cells, expressed the CD127 receptor on their cell membrane, which mediates cell survival via the IL-7-driven homeostatic proliferation, exhibited robust T helper 1 activity and showed the capability to migrate into follicles of lymph nodes and cooperate with B lymphocytes for antibody production. Furthermore, the SARS-CoV-2-specific CD8+ T clone populations were rather heterogeneous and included naïve T cells, terminally differentiated effector T-cells and T memory stem cells [43]. A recent investigation has examined blood specimens from SARS-CoV2-infected patients. Thirty-three of these individuals had been admitted to ICUs due to the severity of the disease, six had been admitted to the hospital with a moderate form of COVID-19 and nine suffered from a mild illness, not requiring hospitalization. This research has shown that elevated numbers of SARS-CoV-2-specific T cells with the capability of undergoing homeostatic proliferation were detectable in patients who resolved the disease. On the other hand, high frequencies of regulatory T cells specific to the SP of this pathogen, as well as a time-dependent increase in levels of activated bystander CXCR4^+^ T cells, were identified in subjects with COVID-19 who died from the infection [40]. A further study has been performed in a small number of patients with mild or moderate forms of COVID-19 who have not been hospitalized and who have recovered from the disease, as well as in some control individuals. The research has examined the responses of both peripheral blood mononuclear cells in general and circulating memory T cells in particular via enzyme-linked immunosorbent spot (ELISpot) analysis in the presence of different pools of peptides belonging to the spike (S) and the nucleocapsid (N) proteins of SARS-CoV-2; it has led to the identification of several immunodominant epitopes in these molecules. The S and N viral peptides tested in this study were not strongly immunogenic, and they could not induce potent T cell responses in all individuals infected by the virus. These antigenic determinants have been demonstrated to stimulate the production of neutralizing antibodies with protective activities even in individuals unexposed to this pathogen. However, the authors have suggested that the design of protective vaccines against SARS-CoV-2 should include a large spectrum of epitopes from different viral proteins, as none of these single peptides were able to promote a strong T cell response [79]. Taking advantage of the results of all available studies in patients with acute HBV and SARS-CoV-2 infection, a dysregulated function of specific T cells has been described for both pathogens. Based on longitudinal immunophenotypic analysis of T lymphocytes, individuals with more severe forms of the disease are characterized by a significant decrease and delay in the emergence of peripheral CD4+ and CD8+ T cells specific to both viruses. Furthermore, the presence of circulating T cells specific to epitopes of both pathogens has not been observed in both HBV and SARS-CoV-2 acutely infected patients with a worse outcome [37]. However, our understanding of the immune pathogenesis of COVID-19 is almost exclusively derived from investigations that have analyzed the immune response in peripheral blood. On the other hand, only a small number of studies have investigated the events occurring in the organs or tissues, mainly targeted by the virus. Furthermore, the available trials have not yet definitively established whether the decreased circulating SARS-CoV-2-specific T cells in patients with severe COVID-19 are undetectable due to their recruitment in the host's tissues, particularly in the lung, or to their exhaustion/deletion. The study methodology used for these studies also may contribute to explaining this lack of understanding. For instance, IFN-γ ELISpot analysis has been able to detect only peripheral Th1 lymphocytes, releasing cytokine IFN-γ. Therefore, technique provides no data about the tissue localization of CD4+ T lymphocytes in the subjects studied. However, the pattern of immune cells in general and of T lymphocytes in the blood may not reflect their spectrum, their distribution or their compartmentalization in the host's organs. This factor adds further difficulty in the interpretation of the results concerning the immune response against SARS-CoV-2, but it has also characterized the study of the immune response against HBV several years ago. Therefore, some studies are longitudinally, and in parallel, examining the immunological profile of circulating or lung-resident CD8+ T cells from patients with COVID-19 of different levels of severity. Szabo et al. studied the activities and the role of T cells, monocytes and macrophages detectable both in the airways and in the blood samples of patients with serious illness by using flow cytometry and scRNA-seq analysis as compared to healthy controls. Furthermore, they correlated the functional features of these immune cells with the age and the disease outcome of these individuals. The research has demonstrated that older patients had lower frequencies of T lymphocytes in the respiratory tract, higher numbers of myeloid cells, such as monocytes and macrophages, persistent pulmonary phlogosis as a worse outcome and an enhanced risk of death than younger individuals, who had more elevated levels of resident T cells in the lungs, a decreased degree of inflammation and better survival rates [80]. Saris et al. profiled the immune responses in the peripheral blood and in the bronchoalveolar lavage fluid (BALF) of 17 patients with COVID-19 who were admitted to the ICU in the late phases of the infection. By using spectral flow cytometry methodology, they studied the classes and the composition of the cells involved in the pathogenesis of the disease. Furthermore, they measured the inflammatory mediators detectable in the blood and BALF. T lymphocytes, mainly CD4+ and CD8+ with an effector memory cells pattern, and macrophages were the most plentiful elements in the BALF. These clones of T cells exhibited levels of the exhaustion marker programmed death-1 that were more elevated than the peripheral blood ones on their cell membrane surface. Decreased frequencies of activated T lymphocytes were found in the plasma, as well as in the BALF in patients with long-term hospitalization (>14 days). Higher levels of inflammatory mediators were observed in the BALF than in the blood. On the other hand, a more elevated number of circulating T lymphocytes was detectable in individuals who died [81]. Furthermore, in their research, Grau-Expósito et al. studied cellular immune profiles in the airways and blood of a total of 46 patients with COVID-19 of different levels of severity (14 symptomatic non-hospitalized cases, 20 mild hospitalized cases and 12 severe hospitalized cases). They reported that a subclass of T lymphocytes expressing the phenotypical features of resident memory T cells (TRMCs) detectable within the lung parenchyma are probably needed to control SARS-CoV-2 diffusion in the host, as well as to prevent the severity of COVID-19. Most TRMCs do not recirculate; therefore, this situation impairs the possibility of analyzing these cells in the blood due to their rapid recruitment into the respiratory tract. In this research, the authors studied circulating virus-specific T cells during the acute phase of infection, describing functional, migratory and apoptotic patterns induced by viral proteins; they associated these profiles with the clinical outcome of these patients. A better prognosis was observed in non-hospitalized individuals, who exhibited elevated serum levels of IL-12, p70 and IL-10 released by SARS-CoV-2-specific CCR7+ T lymphocytes in response to N peptides. On the other hand, T cell responses with IFNγ and IL-4 production were mainly detectable in patients with severe forms of COVID-19 [82]. Grant et al. studied the profile of the immune response in the alveoli of 88 subjects with severe forms of acute respiratory failure, as related to SARS-CoV-2 infection, and required mechanical ventilation; they analyzed BALF specimens via flow cytometry and bulk transcriptomic profiling. The research has demonstrated that the pulmonary alveoli of the patients contained large amounts of T lymphocytes and monocytes/macrophages, and that the macrophages detectable in these spaces were infected with SARS-CoV-2. This event induced alveolar macrophages to produce several mediators with chemo-attractant properties for T cells. The recruitspecificment of these lymphocytes into alveolar spaces was associated with the release of IFN-γ by these populations of T cells. This protein stimulated the synthesis of cytokines by lung macrophages and these mediators, in turn, promoted the enrollment of further T cells and monocytes/macrophages, generating a self-maintaining and persistent inflammatory process in the alveolar spaces of lung tissue [83]. A further study has emphasized that obesity and lymphopenia, which mainly affect CD8+ T lymphocytes, are two predictors of poor outcomes in individuals with SARS-CoV-2 infection and selective CD8 T cells associated with systemic inflammation. In particular, the absolute number of neutrophils was more elevated in patients requiring ICU admission as compared to non-ICU individuals [84]. Overall, all of these studies suggest that subjects with SARS-CoV-2 infection, who mount an antiviral response without an intense inflammatory reaction in the respiratory tract, present a self-limited disease and a better prognosis. On the other hand, individuals with a worse outcome display an exuberant and excessive inflammatory response in the alveolar spaces. These results are in accordance with the previous study by Maini et al. Patients with HBV infection who are able to inhibit the replication of this pathogen present functionally active specific-CD8+ T cells without an associated strong non-specific inflammatory response in the liver and they do not develop severe forms of hepatic injury [57]. On the other hand, in individuals who are unable to control HBV infection, CD8+ T cells, specifically recognizing HBV antigens, are scattered into an intense inflammatory infiltrate, which also includes CD8+ T lymphocytes non-specific to HBV (defined as bystander T cells), monocytes and macrophages. This pattern of response prevents the generation of a protective immune reaction in these patients, who generally undergo a poor clinical course. Therefore, an important goal of therapeutic strategies or vaccination programs in patients with SARS-CoV-2 infection should be the prevention of the intense inflammatory process in the host tissues that is triggered by both viruses. Decreased inflammation is associated with the maintenance of normal CD 8+ T cell functions and better antiviral activities.

## 4. Possible causes of an eventual suboptimal response to vaccination against SARS-CoV-2 and possible strategies to improve it

According to the current knowledge, an optimal response to vaccination with the generation of effective humoral and cellular protection against bacteria and viruses in individuals undergoing this preventive measure depends on a large series of variables. All of these factors, which modulate the immunogenicity of vaccines, have been synthesized in a recent review and are represented by the host's general characteristics, such as age, sex, genetics, comorbidities, preexisting immunity, previous infections, gestational features, microbiota and antibiotics, by the nutritional and behavioral variables of the host, such as body mass index, micronutrients, enteropathy, smoking, alcohol use, exercise and sleep, by environmental factors, such as geographic location and seasonality, and by vaccine-related factors, such as type of vaccine, adjuvant substances, dose, site and schedule of administration [85]. All of these conceptual elements, in association with the results emerging from the prospective/retrospective/observational studies and from reviews about these topics, emphasize the need to differentiate the concept of vaccine efficacy (this word relates to the ability of a vaccine to prevent disease and also pathogen transmission, under ideal and controlled conditions via the comparison of a group of vaccinated people with a group of unvaccinated individuals) and effectiveness (this term refers to how well a vaccine works in the real clinical practice) [86],[87]. A systematic discussion of all of these variables and factors is beyond the scope of this review; therefore, we will only examine some of these factors, i.e., those affecting the immunogenicity of vaccines against SARS-CoV-2. In particular, we will consider several host characteristics, such as age, sex, comorbidities and nutritional status (mainly the intake of micronutrients), as well as some environmental factors, such as geographic location and seasonality. Available reports suggest that the results of the anti-SARS-CoV-2 vaccination strategy may be suboptimal in some groups of subjects worldwide, and that the use of this measure may not lead to an effective immunization against this pathogen in these individuals [88]. The following general and clinical characteristics may have an important impact on the development of an effective antiviral immune response after the administration of vaccines against SARS-CoV-2 in patients exhibiting these features: i) older age [89], ii) malignant chronic diseases (i.e., tumors) [90], iii) non-malignant chronic diseases [91],[92] and iv) organ transplantation [93]; they are all clinical conditions that significantly decrease the efficacy of vaccination [94].

In particular, aged individuals are at increased risk of infections and present higher rates of comorbidities or more severe forms of the disease as compared to younger individuals. Despite some uncertainties, there is consensus that standard vaccines against some pathogens are less immunogenic and efficient in older versus younger adults, and that elderly subjects generally exhibit a reduced response to these prophylactic measures in the real world. Therefore, vaccination coverage is not yet satisfactory for some of these vaccines [89]. For instance, a recent meta-analysis has investigated the immunogenicity of the influenza vaccine in elderly people and its association with the real world. The seroprotection rate and seroconversion rate of older adults were lower as compared to those in younger individuals for A/H1N1 and B/Victoria, while the two age groups had similar antibody responses for A/H3N2. The antibody responses to vaccines were not significantly associated with real-world vaccine effectiveness, indicating that antibody response might not fully reflect the vaccine effectiveness of A/H3N2 [95]. Therefore, further strategies to improve the response to vaccines in aged people and vulnerable groups, who display a suboptimal magnitude and quality of immune activation after the use of this preventive measure, are needed. This strategy also has important implications in the evaluation of the short- and long-term efficacy and effectiveness of vaccines against SARS-CoV-2, also as related to the type of preparation that is inoculated. In their study, Collier et al. have shown that about half of the individuals over the age of 80 who were enrolled in the trial presented both suboptimal neutralizing antibody and decreased T cell responses after the first dose of BNT162b2, as compared to younger subjects. The authors hypothesized that the reduced activity of the immune system in its humoral and cell compartments that was detectable in a proportion of these aged people might be due to several factors, such as a lower concentration (quantity), a lower affinity (quality) of antibodies resulting from B cell selection, less CD4+ T cell help or a combination of some or all of these factors [96]. In their population-based study, Cerqueira-Silva et al. investigated the influence of age on the effectiveness and duration of protection of the Vaxzevria and CoronaVac vaccines (a viral vector and an inactivated virus vaccine, respectively), suggesting a high efficacy in individuals up to 79 years of age [97]. A very recent trial in France has reported that mRNA-based vaccines have been able to decrease the risk of severe forms of SARS-CoV-2 infection in more than 1.4 million people aged 75 years and over. The research has shown a significant reduction in the risk of death due to COVID-19 (about 91%) more than a week following the second dose in the vaccinated subjects. However, the observational period in these individuals after vaccination against COVID-19 has been rather short, since this program was started on December 27, 2020, whereas the end of the follow-up was on March 20, 2021. Therefore, to date, no definitive data results on the long-term effectiveness of this preventive measure are available [98]. It is well known that elderly people display an age-related functional decline in cellular immunity. This phenomenon is known as immunosenescence, and it makes these individuals less able to mount an effective cellular immune response to vaccination, making these people more vulnerable to the risk of viral diseases related to morbidity and mortality and less prone to responding to anti-viral vaccination strategy [99]. In addition, in older people, a status of chronic inflammation known as “inflammaging” that causes harmful effects on their immune function has been observed. Inflammaging is associated with pathological conditions, including cancer, arteriosclerosis and neurodegenerative diseases, such as Alzheimer's and Parkinson's disease. All of these comorbidities increase the risk of viral infections and may promote a serious progression of their course [100]. Furthermore, malignancies are associated with an increased risk of death (about 13% 30-day all-cause mortality and 10–30 times higher than one observed in the general population) from COVID-19 [101]. Current evidence has suggested a decreased overall antibody and T cell response following two doses of the BNT162b2 COVID-19 vaccine relative to the immunocompetent study controls [90],[102]. The available investigations have shown the following in subjects with neoplasms: a) anti-SARS-CoV-2 adaptive T cell immunity is weaker than immunity developed towards common viruses; b) the characterization of anti-SARS-CoV-2 immunity is not sufficiently insured by the monitoring of specific immunoglobulin and assays, so specific T cell responses must be monitored; c) the memory T cell response is not impaired by SARS-CoV-2 infection [103],[104]. Also, non-malignant chronic diseases and organ transplantation are clinical conditions significantly decreasing the efficacy of vaccination. A decreased efficiency in cell and humoral immune responses has been recognized and described in these groups of individuals [91]–[94]. Overall, all of these situations are associated with a suboptimal response to anti-SARS-CoV-2 vaccination, and with a decreased probability of reaching adequate protection against this pathogen, including proper functioning of the innate and adaptive arms of the immune system. Similar mechanisms are involved in the genesis of all of these pathological conditions. The results of these trials strongly suggest a more cautious use of the terms vaccination and immunization. These two words are often used interchangeably in non-specialist settings, including in international news media and on social media platforms, but this practice is inappropriate and likely to generate considerable confusion and misunderstandings by not only the general population, but also among non-health personnel who have the task of making decisions on public health [105]. Vaccination may not lead to the effective immunization of individuals receiving this procedure. As reported above, some predictors of a suboptimal response to vaccines have been reviewed, but several factors and mechanisms associated with the unsatisfactory immune response remain unclear or not fully elucidated [36],[106]. Further studies are required to obtain more detailed knowledge on the extent, efficacy and longevity of the adaptive humoral and cellular immune responses elicited by the causative agent of COVID-19 after infection or after vaccination against this pathogen, and of the cooperative interplay between these two components [39].

## 5. Possible role of nutrients in modulating the response of distinct immune cells to vaccination against SARS-CoV-2

Starting from all of the above-reported considerations, it is conceivable that the decrease in the efficacy and effectiveness of anti-SARS-CoV-2 vaccines in aged and immune-compromised patients may be counteracted. Some transdisciplinary investigations are currently in progress, focusing on micronutrients and nutritional factors, including long-chain omega-3 polyunsaturated fatty acids (n-3 PUFAs), such as α-linolenic acid (ALA or 18:3, ω3), eicosapentaenoic acid (EPA or 20:5, ω3) and docosahexaenoic acid (DHA or 22:6, ω3), as well as fat-soluble compounds, such as vitamins A, D and E. Current evidence suggests that these dietary elements may contribute to regulate the function of the immune system in its innate and adaptive arms, and to modulate some crucial host defensive processes, such as inflammation. Furthermore, the studies suggest that these nutritional factors may also strengthen the immune responses of living organisms to vaccination and prolong their duration. In particular, we will analyze the available results from studies on this topic and subdivide them into distinct subgroups: i) experimental studies in animal models, ii) conceptual and perspective reports on mankind and iii) trials performed on humans.

### 5.1. Experimental studies in animal models

Since several years ago, a large series of experimental investigations have analyzed the role of some essential dietary elements in modulating the function of the immune system and their effects on the immune response after the administration of vaccines in different species of animals (mainly birds and mammals). Several nutrients have been examined in these studies, including n-3 PUFAs, such as ALA, EPA and DHA [107],[108], as well as fat-soluble vitamins (vitamins A, D and E) [109]–[111]. According to available studies, the normal activity of the immune system in living organisms also depends on the coordinated and tightly regulated actions of all of these essential dietary elements. Supplementation of the feed with high levels of n-3 PUFAs has been tendentially associated with a better humoral immune response in animals enrolled in several trials, but the global effect is influenced by several factors, such as the species of living beings considered and the type of vaccines used [112]–[114]. Conjugated linoleic acid has been reported to exhibit immunoenhancing properties both in young and old mice. A high intake of this PUFA in the feed of both groups displayed a significantly higher splenocyte proliferation and IL-2 production [115]. Nevertheless, elevated dietary n-3 PUFA intake has also been associated with a decreased production and release of immunoglobulins, as well as with a modulation in the phenotype of T cells, with a shift from Th2 lymphocytes subclasses to T helper cell type 1 (Th1) ones in the organisms recruited in these studies [113]. However, a series of experimental investigations has shown that an enriched n-3 PUFA composition of feed may improve the defensive role of humoral and cellular responses after vaccination against different pathogens such as viruses or bacteria in male mice and broiler chickens [107],[108]; other trials have not confirmed these results. Some studies have not found an association between elevated levels of PUFA intake and increased production of immunoglobulins in vaccinated animals [116]. Furthermore, fat-soluble vitamins A, D and E are also involved in the proper function of the immune system in animals, and deficient levels of these micronutrients have been associated with the impairment of innate and adaptive (in its humoral and cell component) immune responses against several types of pathogens in a few trials which have been carried out in different species of animals [117],[118]. Penkert et al. reported that mice with a shortage of vitamin A displayed the production of elevated amounts of viral antigens and highly up-regulated expression of pro-inflammatory cytokine/chemokine in nasal tissues. These vitamin A-deficient animals have experienced the persistence of the virus in the upper and lower respiratory tract. A long-lasting viral infection was found to maintain a harmful pro-inflammatory stimulus even in the presence of T-regulatory cells. The authors have concluded that a fast and strong immune response against the virus may instead be desirable so that the control of the pathogen may prevent this persistent trigger, leading to cytokine release [119]. Studies in animal models have also shown a relationship between vitamin D deficiency and an increased risk of autoimmune diseases in immunocompetent mice. Furthermore, supplementation with different doses of this micronutrient in animals suffering from glucocorticoid-induced immunosuppression has been able to improve their immune function via stimulation of T cell proliferation and elevation of IL-2 production [120]. Influenza virus vaccination in vitamins A- and D-deficient mice has been also associated with decreased frequencies of virus-specific CD8+ T lymphocytes [121]. The supplementation with vitamins A and D, at the time of immunization with a cold-adapted influenza virus vaccine, has been demonstrated to restore, in a dose dependent-manner, the impaired production of immunoglobulins detectable in animals with a shortage of both micronutrients [122]. Vitamin E also represents a micronutrient essential for the normal activity of the immune system in animals. For instance, supplementation with this fat-soluble compound has also been demonstrated to improve the immune response in old mice. The suppression of prostaglandin E1(2) synthesis is one of the mechanisms by which this micronutrient exert its activity [123]. Age-associated decrease in T cell signaling in old mice represents one of the factors, preventing the effective generation of immune synapses among T cells and APCs. In particular, CD4+ T lymphocytes detectable in aged-mice exhibit a lower number of immune synapses than those observed in young mice. The percentage of CD4+ T cells establishing a functional immune synapse with APCs is enhanced in vitro and in vivo by VE supplementation [124],[125]. Recently, vitamins A, D and E have been used as influenza vaccine adjuvant components. Among all of these formulations, the one containing VE represents one of the most promising combinations [126]. The supplementation with different forms of this micronutrient increases the host's immune response to vaccination. Mice fed with a different mixture of vitamin E isomers, including tocotrienol-rich fraction, alpha-tocopherol and delta-tocotrienol, displayed an improved immune response to the tetanus toxoid vaccine via the promotion of a Th1 response [127].

### 5.2. Trials performed in humans

The immunoregulatory and anti-inflammatory role of diets enriched with n-3 PUFAs or fat-soluble vitamins A, D and E have already been well recognized and described some years ago also for several human pathological conditions, such as infectious and autoimmune diseases [128],[129]. A large number of clinical studies and reviews has shown that all of these dietary factors may exert modulatory activities on the function of several key cells and mediators involved in orchestrating a proper immune response in its innate and adaptive components. Furthermore, reported investigations have analyzed the effects of PUFAs and fat-soluble vitamin deficiency on human health [130]–[134]. Taking advantage of all of these studies, it is crucial to analyze the status of n-3 PUFAs and fat-soluble vitamins in the global population. This approach may contribute to understanding whether the use of a diet enriched with these nutrients in deficient people may be a useful strategy to improve the immune system reactivity against SARS-CoV-2 infection, as well as the immune response to vaccination toward this pathogen, mainly in individuals with persistent immunosuppression, such as aged subjects. Among the PUFAs, omega-3 (n-3) and omega-6 (n-6) families exert important regulatory functions in human health; these classes of metabolites generally exhibit opposing effects. n-6 PUFA-derived lipid mediators promote inflammation, platelet aggregation and vasoconstriction. On the other hand, n-3 PUFA-derived lipid mediators inhibit inflammation and platelet aggregation and induces vasodilation. Elevated consumption of n-6 PUFAs and low intake of n-3 PUFAs is associated with the development of many current diet-related persistent diseases. Although the role of n-3 PUFAs in modifying the risk of a wide range of human diseases is now known, it is not yet well established what levels of these circulating essential dietary elements can be defined as normal. However, some investigations from North America have detected in healthy adults the following serum concentrations of LNA (alpha-linolenic acid), EPA, and DHA: 54.4 ± 25.1 µmol/L, 40.3 ± 28.3 µmol/L, and 88.8 ± 36.8 µmol/L, respectively [135],[136].

On the basis of this knowledge, the n-6 PUFAs to n-3 PUFAs ratio has to be considered as a crucial factor for human health. An elevated n-6 PUFA/n-3 PUFA proportion exists in modern diets as compared to the ones used several decades ago [137]. According to the Global Health Data Exchange Global Burden of Disease (http://ghdx.healthdata.org/gbd-results-tool, data extraction, March 6, 2021), the age-standardized rate per 100,000 population prevalence of vitamin A deficiency (normal serum retinol level ranging from 20 to 60 µg/dL) across the world has progressively decreased from 1990 to 2019. However, although only a small series of data about the status of vitamin A in the general population across the globe is available, reduced serum levels of this micronutrient are most commonly detected in children and in pregnant women, as well as in males and low-income countries [138]. However, the study has shown that, excluding the peak vitamin A deficiency seen in children, the circulating amounts of this micronutrient tended to be lower with the increase in the enrolled people's age [139]. Current studies have shown that an elevated prevalence of vitamin D deficiency (with serum levels < 12 ng/mL) or insufficiency (with serum levels ≤ 20 ng/mL) exists worldwide, as well as in regions localized at low latitudes, although the UVB radiation is detectable in these countries and generally considered sufficient to prevent the development of this condition. Suboptimal serum levels of this micronutrient has been described even in high-income nations, where programs of vitamin D supplementation have been implemented since several years ago. Furthermore, deficiency or insufficiency of this organic molecule is detectable not only in aged people (reaching a prevalence of about 15–16% and 50–60%, respectively, in Europe, as well as 4% and 35% in North America and China), but also in younger people (with a prevalence of about 10–15% and 30–40%, respectively). These percentages are variable, depending on the sex of individuals and the season of year considered [140]. The results concerning the possible effects of vitamin D deficiency on the capability of individuals to mount an effective serological response after vaccination are not univocal. Some trials have shown that the serum status of this micronutrient is not associated with a serological response to the influenza virus vaccine also in individuals older than 50 years [141]. Furthermore, a systematic review and meta-analysis have analyzed the possible influence of vitamin D deficiency on the seroprotection rates and seroconversion rates after influenza vaccination with conflicting results. However, no overall association has been described between the shortage of this micronutrient and immunogenic response to this preventive measure. Decreased seroprotection to influenza A virus subtype H3N2 (A/H3N2) and B strain has been observed in vitamin D-deficient individuals relative to patients with normal serum levels of this dietary element [142]. According to the recommendations by the Food and Nutrition Board of the Institute of Medicine, vitamin E deficiency corresponds to circulating α-tocopherol concentrations <12 µmol/L, whereas a broadly accepted cut-off point for optimal vitamin E status is ≥30 µmol/L [143],[144]. Only a small number of studies have investigated the status of vitamin E in people living in distinct areas of the world. Deficiency rates of this micronutrient present as considerable among the different classes of ages included in the available investigations. The highest frequencies of vitamin E concentrations (<12 µmol/L) have been detected in newborns and children up to 12 years, whereas only 21% of older adults (>50 years) had desirable serum levels (≥30 µmol/L) [145],[146]. Based on the results from the available studies, maintaining optimal concentrations of the above-mentioned nutrients in the hosts' serum and tissues, including n-3 PUFAs and fat-soluble vitamins, may represent a desirable goal. All of these dietary factors acting in tandem have demonstrated the ability to remodulate, rebalance and improve the function of the immune system in its innate and adaptive components, preventing the development of some defensive but potentially harmful effects, such as the emergence of uncontrolled inflammation. These beneficial activities have been observed in immunosuppressed individuals, including elderly people [147]–[151], as well as in individuals with cancer [151]–[153] and autoimmune diseases, although some studies have not confirmed these results.

## 6. Future perspective

The large number of studies presented in the previous sections of this paper provide a rationale for the development of strategies useful for counteracting SARS-CoV-2 infection. These approaches may be pursued via two key pathways:

1) Increasing and improving the effects of vaccination against this pathogen.

2) Modulating and rebalancing the function of the immune system in innate and adaptive arms.

The current knowledge of other viruses' immunopathogenesis, such as that of HBV, suggests that patients with acute infection who are able to effectively control the viruses, mount a multi-specific and polyclonal immune response against a large series of epitopes belonging to different viral proteins [155]. In particular, the HBV nucleocapsid represents a key component of the virion, as some of its antigenic determinants stimulate the generation of specific CD4+ T lymphocytes. This event is crucial in the induction of a protective immune response against this pathogen. CD4+ T clones specific to the nucleocapsid epitopes cooperate with CD8+ T cells and activate their ability to release antiviral cytokines or exert direct cytotoxic function against the host's infected hepatocytes, causing their damage and necrosis [73]. The reduced generation of CD4+ T cells specific to nucleocapsid epitopes or their dysregulated function is associated with the impairment of antiviral immune response, leading to an increased risk of HBV persistence [72],[74]. Starting from all of these considerations, it is conceivable that similar or common immunopathogenic mechanisms may function during the development of infections linked to both pathogens. Therefore, it can be hypothesized that this concept could be useful in the design and preparation of new vaccines against SARS-CoV-2. This type of biological preparations should include a well-selected spectrum of epitopes that belong to different viral proteins and are capable of eliciting multi-specific, polyclonal and strong immune and humoral responses. The design of vaccines stimulating the generation of CD4+ and CD8+ T lymphocytes specific to the SARS-CoV-2 nucleocapsid protein may be of particular relevance in inducing an adequate protection against COVID-19. A recent experimental study has assessed, in an animal model, whether the use of a vaccine not eliciting responses directed toward the SP of SARS-CoV-2 may be an effective strategy. Vaccination of K18-hACE2 mice and Syrian hamsters with a human adenovirus type 5 vector expressing the SARS-CoV-2 nucleocapsid protein has promoted protective immunity in these organisms. A decreased weight loss and viral load have been found in both types of animals [156].

The current evidence, suggesting a beneficial role of n-3 PUFAs and some fat-soluble vitamins in the modulation and rebalancing of innate and adaptive immune responses, provides a strong rationale for the use of these nutrients as a complementary treatment or preventive or therapeutic strategy during the SARS-CoV-2-related pandemic [157],[158]. Several n-3 PUFA metabolites, defined as resolvins, protectins and maresins, as well as vitamins A, D and E, have been shown to display protective functionality in several human illnesses, such as autoimmune or infectious pathologies, including acute viral infections [159]. These molecules modulate the magnitude of inflammatory events that are detectable in these diseases by exerting pro-resolving activity in these processes [160]. Therefore, all of these nutrients and their metabolites contribute to controlling the severity of human pathological conditions. Several experimental studies and reviews have described the function and activity of these dietary elements during SARS-CoV-2 infection [161],[162].

According to some preliminary investigations, supplementation of COVID-19 patients with moderate dosages of n-3 PUFAs may provide beneficial effects in the control of inflammation-mediated clinical manifestations [163],[164]. Although the results of these trials are promising, to date, no definitive conclusions have been obtained. Therefore, further studies are needed to definitively clarify this topic. Vitamin A acts by modulating several elements of the immune response. In particular, this micronutrient has a crucial role in regulating the development of B-cells and the synthesis of IgA [165]. These antibodies are involved in the protection of the host's mucosal membranes against pathogens. These structures in the respiratory tract represent the most important route for SARS-CoV-2 entry. A very recent study has examined how the RNA vaccine (BNT162b2 COVID-19 vaccine) generates specific immunity at mucosal sites. This type of vaccination protocol, after the second dose, induced a low IgA concentration in saliva, whereas neutralizing antibody levels (IgG class) were more elevated in serum. Therefore, this study shows that mucosal immunity is poorly stimulated and could not control virus infectivity [166]. Proper intracellular and serum concentrations of vitamin A are necessary for the synthesis of protective IgA amounts for SARS-CoV-2. Vitamin D status also represents an important and well-known parameter for the normal function of the immune system in humans. Some epidemiological and clinical investigations have reported that this micronutrient exerts significant activities in counteracting virus-related infections of the respiratory tract [167],[168], whereas its deficiency is associated with a more elevated risk of admission to ICUs [169] and higher mortality rates in individuals suffering from more severe forms of pneumonia [168],[170]. According to this evidence, several studies have been performed with the following purposes: i) to investigate the possible association between vitamin D status and risk of ICU admission rate, ventilator support requirement, length of hospital stay and in-hospital mortality in patients with COVID-19, and ii) to analyze the effects of this micronutrient supplementation on the above-mentioned factors in SARS-CoV-2-infected subjects. The interest of the scientific world in assessing the possible beneficial effects of this fat-soluble compound has now led to about 50 meta-analyses. Some of these works suggest that vitamin D deficiency/insufficiency increases susceptibility to COVID-19 and severe forms of this infectious disease, with longer in-hospital stays, more elevated requirements for ICU admission and higher mortality rates [171],[172]. Furthermore, supplementation of SARS-CoV-2-positive patients with this micronutrient decreases the risk of a poor outcome and improves their prognosis [173],[174].

On the other hand, other meta-analyses have found that no significant relationship exists between vitamin D deficiency and risk of COVID-19 severity, such as the in-hospital mortality, need for ICU admission and duration of in-hospital stay [175]. Furthermore, intake of this fat-soluble compound has produced no improvement in outcomes for hospitalized patients [176]. Several causes may contribute to explaining these discrepancies, such as differences in the design and endpoints of different studies considered, type of enrolled patients and dosage of vitamin D administered to recruited individuals, as well as the length of the treatment period. Vitamin E is also required for the normal function of cells and the humoral immune response. In particular, this micronutrient may counteract the age-associated decline in cellular immune response, as it contributes to preserving Th1 function. The reduced vitamin E-mediated production of prostaglandin E_2_, a T cell suppressive factor, is associated with the enhanced release of IL-2 and with the increased proliferation of T cells [147],[177]. According to this evidence, some randomized studies have explored the possible antiviral role of this dietary factor. Although the available studies differ in their design and have enrolled a small number of patients, vitamin E supplementation has been shown to induce high rates of serum HBeAg loss with HBeAb seroconversion, with transaminases normalization and HBV-DNA clearance, both in adults and in children [178]–[180]. On the other hand, in their study, Dikici et al. showed no beneficial effects in HBeAg-positive pediatric patients who were treated with vitamin E [181]. The reasons explaining these discrepancies are not well known. Nevertheless, Dikici et al. used a lower vitamin E dosage with a shorter time of supplementation and a more restricted follow-up period after the end of treatment (100 mg/day, 3 months of treatment, and 6 months of follow-up) as compared to the study by Gerner (200 to 600 IU/day depending on body weight, 6 months of treatment and 12 months of follow up) and to the trial by Fiorino and colleagues (15 mg/kg per day, 12 months of treatment and 12 months of follow up) [180]–[182]. High rates of HBV-DNA loss and HBeAb seroconversion have mainly been observed at the end of treatment or during follow-up. These results strongly suggest that the modulatory activities of vitamin E and, probably, vitamins A and D and PUFA require not only a correct dosage of these dietary factors, but also an adequate period to develop, as they have a broad spectrum of complex regulatory actions involving both innate and adaptive immune responses. Therefore, based on all of these considerations, these micronutrients should not simply be considered as supplements, but as real drugs, taking into account their possible beneficial or harmful effects [157],[158].

The use of all of these nutrients might exert beneficial effects, mainly in immune-compromised individuals, both by improving the response rate to SARS-CoV-2 vaccination and by attenuating the degree of inflammatory events that are detectable during the development of COVID-19 via modulation of Th1/Th2/Th17 imbalance and reduction of the ratio between the pro-inflammatory and anti-inflammatory cytokines. Additional trials are required to improve our understanding of these topics, with the purpose of applying the knowledge acquired from these studies for the preparation of new types of vaccines and treatments to effectively counteract SARS-CoV-2.
